# How the Results of a Randomized Trial of Catheter-Directed Thrombolysis versus Anticoagulation Alone for Submassive Pulmonary Embolism Would Affect Patient and Physician Decision Making: Report of an Online Survey

**DOI:** 10.3390/jcm8020215

**Published:** 2019-02-07

**Authors:** Bedros Taslakian, Clayton Li, Samuel Z. Goldhaber, Kathryn Z. Mikkelsen, James M. Horowitz, Christopher Kabrhel, Geoffrey D. Barnes, Akhilesh K. Sista

**Affiliations:** 1 Department of Radiology, NYU Langone Health, New York, NY 10016, USA; Clayton.Li@nyulangone.org (C.L.); Akhilesh.Sista@nyumc.org (A.K.S.); 2 Division of Cardiovascular Medicine, Brigham and Women’s Hospital and Harvard Medical School, Boston, MA 02115, USA; sgoldhaber@bwh.harvard.edu; 3 North American Thrombosis Forum, Brookline, MA 02445, USA; kmikkelsen@natfonline.org; 4 Department of Medicine, NYU Langone Health, New York, NY 10016, USA; James.Horowitz@nyulangone.org; 5 Center for Vascular Emergencies, Department of Emergency Medicine, Massachusetts General Hospital, Boston, MA 02114, USA; CKABRHEL@PARTNERS.ORG; 6 Frankel Cardiovascular Center, University of Michigan, Ann Arbor, MI 48109, USA; gbarnes@med.umich.edu

**Keywords:** pulmonary embolism, submassive, survey

## Abstract

The purpose is to investigate how the outcomes of a randomized controlled trial (RCT) of catheter-directed thrombolysis (CDT) versus anticoagulation alone for acute submassive PE would affect clinical decision-making. An online survey was sent to the Pulmonary Embolism Response Team Consortium members and the North American Thrombosis Forum members. Participants rated their preference for CDT on a 5-point scale in 5 RCT outcome scenarios. In all scenarios, subjects in the CDT group walked farther at 1-year than those in the anticoagulation group. A total of 83.3% of patients and 67.1% of physicians preferred CDT (score > 3) if it improved exercise capacity and did not increase bleeding. In every scenario, patients scored CDT higher than physicians (*p* < 0.05 for each). Bleeding and clinical deterioration were independently associated with the mean score. Patients’ age, gender, and history of PE did not influence CDT scores (*p* = 0.083, *p* = 0.071, *p* = 0.257 respectively). For patients, 60% > 60 years, 65.5% < 60 years, 57.1% of men, and 66.3% of women preferred CDT across scenarios. In conclusion, the majority of respondents would choose CDT if it improves long-term exercise capacity and does not increase bleeding. Patients appear to accept a higher bleeding risk than physicians if CDT improves long-term exercise capacity.

## 1. Introduction

The optimal management of submassive pulmonary embolism (PE) is uncertain. Submassive PE is associated with a higher rate of clinical deterioration and mortality compared to low-risk PE when treated with anticoagulation alone [1,2,3,4]. Survivors may also suffer from a long-term “post-PE syndrome” (PPS) and chronic thromboembolic pulmonary hypertension (CTEPH), characterized by decreased exercise tolerance and quality of life [5,6]. Although anticoagulation alone is the current standard of care, it does not adequately protect against these outcomes. In a recent randomized controlled trial (RCT), there was a 5.6% rate of clinical deterioration within 7 days and a 3.2% 30-day mortality rate in patients with submassive PE who were treated with anticoagulation alone [4]. A meta-analysis of randomized trials demonstrated a survival benefit when systemic thrombolytic therapy is used in patients who present with submassive PE [7]. However, there was a much higher risk of major bleeding compared with anticoagulation alone. In the PEITHO trial, there was a 11.5% major and a 2% intracranial bleeding rate between randomization and day 7 in the systemic thrombolysis group [4]. Therefore, catheter directed-thrombolysis (CDT) has emerged as a promising therapy that has the potential to improve short and long-term outcomes. Targeted delivery of a low-dose thrombolytic drug into the clot through a multi-side hole catheter could mitigate the risk of major bleeding complications while achieving better drug effectiveness [8]. However, CDT’s efficacy and safety have been inadequately evaluated [1,2,9,10,11].

Three prospective studies (ULTIMA, SEATTLE II, PERFECT registry) have studied CDT as a therapy for submassive PE [12,13,14]. ULTIMA, which included 59 patients, was the only randomized trial comparing CDT with anticoagulation. It showed that CDT is superior to anticoagulation alone in improving RV dilation at 24 h, without an increase in bleeding complications. However, the study was underpowered to assess short and long-term clinical outcomes of CDT and the risk of development of PPS or CTEPH. A recent retrospective evaluation of 105 patients with massive and submassive PE showed significant improvement in right/left ventricular ratio at 24–48 h in patients who were treated with catheter-directed therapy compared to anticoagulation alone [15]. A survey of physicians who treat submassive PE suggested a predilection for CDT despite equivocal guidelines and the lack of rigorous studies, further supporting the need for a randomized clinical trial of CDT versus anticoagulation alone [16].

Based on the recommendations of a multidisciplinary panel of experts, an application for a randomized controlled trial (RCT) comparing CDT to anticoagulation for the treatment of submassive PE was submitted to the National Heart, Lung, and Blood Institute (NHLBI) to address this data gap [2]. Because the primary endpoint for this proposed trial evaluates exercise capacity at 1 year using the 6-minute walk test, we sought to understand how physicians and patients would weigh long-term positive trial results against varying risks of bleeding and clinical deterioration. Surveys were sent to members of the Pulmonary Embolism Response Team (PERT) Consortium and the North American Thrombosis Forum (NATF) communities, representing physicians and patients, respectively. 

## 2. Materials and Methods

From 1 September to 20 September 2017, a link to an electronic survey (via surveymonkey.com) entitled “How would the PE-TRACT final outcomes affect your decision making for treatment of submassive PE?” was sent to physician members of the PERT Consortium (http://pertconsortium.org). A similar electronic survey written for laypersons was sent to members of the NATF community (https://natfonline.org) who are either patients who experienced venous thromboembolism (VTE) or families and friends of patients. The surveys were closed on 15 October 2017, and the results were analyzed.

After collecting demographic information, both questionnaires presented 5 scenarios of potential outcomes of an RCT comparing CDT with anticoagulation versus anticoagulation alone for the treatment of submassive PE (Table 1). In all 5 hypothetical scenarios, subjects undergoing CDT had a longer 6-minute walk distance at one year (better exercise tolerance) than those treated with anticoagulation alone. Then, each scenario presented varying rates of 30-day clinical deterioration and 7-day major and intracranial bleeding risk in the 2 groups. In the physician survey, clinical deterioration was defined as cardiac arrest, systolic blood pressure < 90 mmHg for 15 min or greater, vasopressor support, or respiratory decompensation (endotracheal intubation) [4]. Respondents were asked to rate their likelihood of choosing CDT on an ordinal scale of 1–5 (1 (“never”), 2 (“almost never”), 3 (“occasionally”), 4 (“almost always”), and 5 (“always”)). Scores of 4 and 5 indicated a preference for CDT. 

The mean scores with 95% confidence intervals (CIs) were used to compare CDT preferences between physicians and patients, whereas the median scores were used to measure the central tendency for the following reasons: (1) The numbers derived from Likert scales represent ordinal responses. (2) The non-normal distributions of response data can result in a mean score that is not a helpful measure of the data’s central tendency [17,18]. For the purpose of the analysis, the CDT scores were converted into a binary outcome: A score > 3 was considered to represent a preference for CDT.

To assess the relative impact of bleeding risk and clinical deterioration on the scores provided by physicians and patients, two surrogate variables were created. The bleeding and clinical deterioration risks associated with CDT relative to anticoagulation alone were captured in an ordinal variable for bleeding risk and a binary variable for clinical deterioration. A numeric variable for bleeding assumed the value 1 when the treatment options were assumed to have equivalent bleeding risk (scenario 1) and the value 2 or 3 when the bleeding risk of CDT was respectively assumed to be 2× (scenarios 2, 3) or 4× (scenarios 4, 5) higher than that for anticoagulation group. 

Ordinal scores from the 5 scenarios were compared using paired-sample Wilcoxon signed ranks tests. The percentage of participants showing a preference for CDT was compared using the McNemar test for paired binomial proportions. An exact Mann-Whitney test was used to compare patients’ and physicians’ ordinal scores, and the Fisher exact test was used to compare CDT preferences between physicians and patients. Multivariate regression was used to assess the impact of bleeding and deterioration risk on CDT preference. Least squares regression was used to model the ordinal score, and logistic regression was used to model CDT preference. A sample size calculation was performed post hoc using an online sample size calculator (https://www.surveymonkey.com/mp/sample-size-calculator) to evaluate whether there was a sufficient number of physician and patient respondents. All statistical tests were conducted at the 2-sided 5% significance level using SAS 9.3 software (SAS Institute, Cary, NC, USA). *p* values < 0.05 indicated significant findings.

## 3. Results

**Figure 1:**
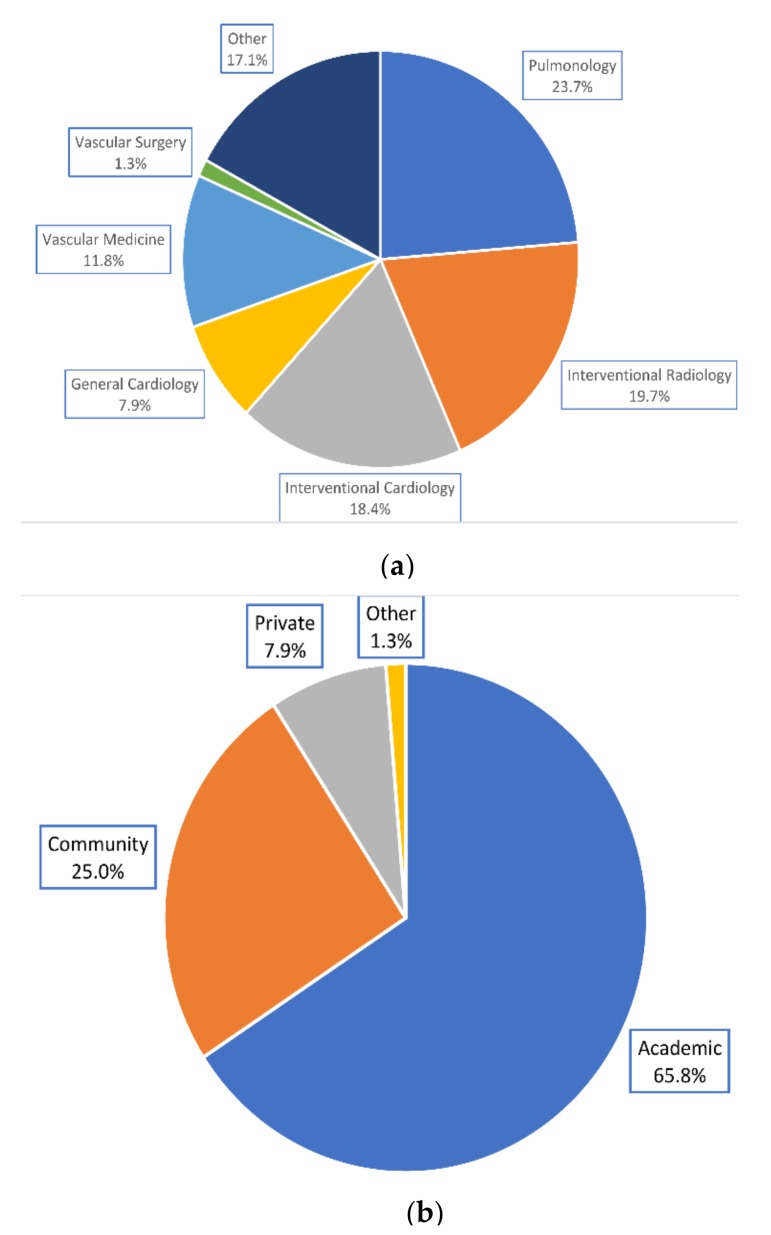
Physicians’ demographics. (**a**) Specialties represented by physicians. (**b**) Practice settings reported by physicians.

Of 76 respondents to the PERT Consortium questionnaire, the majority were pulmonologists, interventional radiologists, and interventional cardiologists, and practiced in an academic hospital (Figure 1). Seventy-two (94.7%) practiced in the United States, 2 (2.6%) in Canada, 1 (1.3%) in Argentina, and 1 (1.3%) in Brazil.

**Figure 2:**
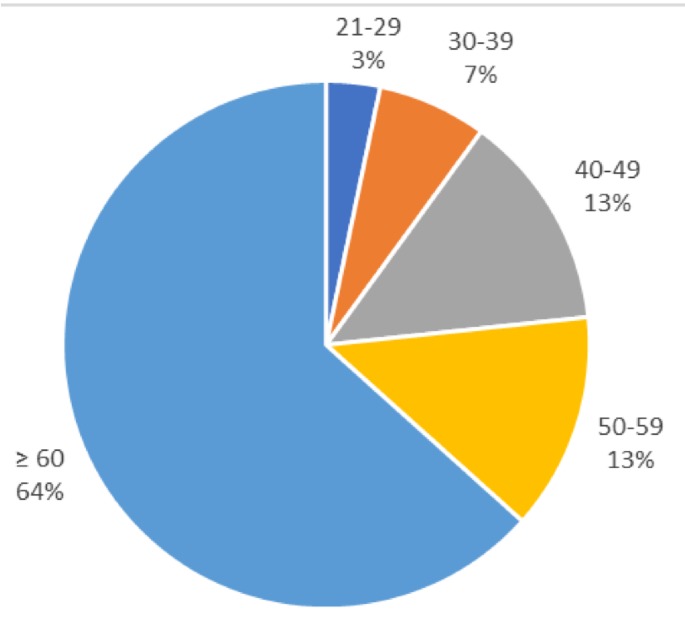
Percentage of patients within each age group.

Of 30 respondents to the NATF questionnaire, 16 (53.3%) experienced PE previously, and 16 (53.3%) were female. The majority of respondents (19; 63.3%) were older than 60 years of age (Figure 2). 

**Figure 3:**
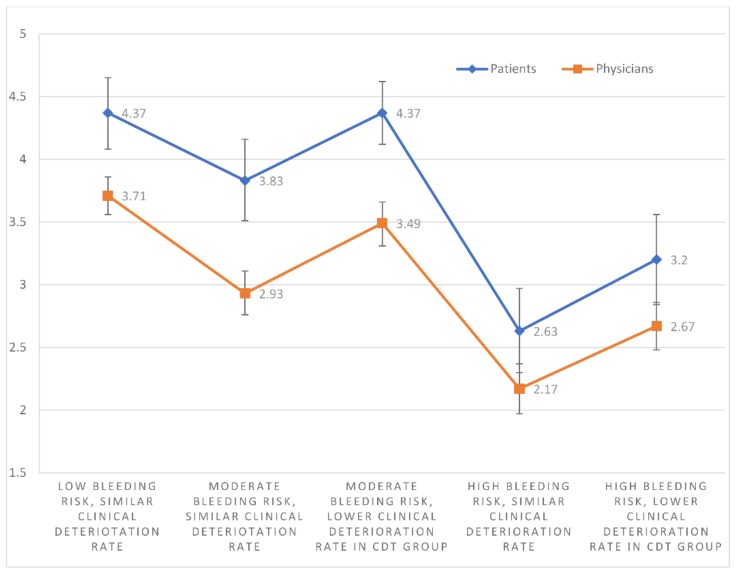
The mean CDT scores with 95% confidence intervals of physicians and patients for each individual scenario.

In each of the 5 scenarios (numbered S1–S5), patients scored CDT significantly higher than physicians (mean CDT scores: S1, 4.37 vs. 3.71 (*p* < 0.001); S2, 3.83 vs. 2.93 (*p* < 0.001); S3, 4.37 vs. 3.49 (*p* < 0.001); S4, 2.63 vs. 2.17 (*p* = 0.012); and S5, 3.20 vs. 2.67 (*p* = 0.003)); (Figure 3). The median patient CDT scores were 5, 4, 4, 3, and 3 for scenarios 1–5, respectively. By comparison, the median physician CDT scores were 4, 3, 4, 2, and 3, respectively. Given a population size of 1346 PERT consortium and 4500 NATF members, to provide an 80% confidence level and an 8% margin of error, 62 physicians and 64 patients would need to respond. An 8% margin of error ensures no overlap between the confidence intervals of the patient and physician mean scores in any of the scenarios.

Patients scored CDT significantly higher in S1 than S2 (*p* = 0.002), S4 (*p* < 0.001), and S5 (*p* < 0.001). Mean CDT scores were higher in S2 than S4 (*p* < 0.001) and S5 (*p* = 0.009). Mean CDT scores were higher in S3 than S2 (*p* = 0.004), S4 (*p* < 0.001), and S5 (*p* < 0.001). The mean CDT score in S5 was also greater than in S4 (*p* = 0.003). S1 and S3 scores were not significantly different (*p* = 1.000) (Figure 3).

Physician responses showed the same pattern. They scored CDT significantly higher in S1 than S2 (*p* < 0.001), S4 (*p* < 0.001), and S5 (*p* < 0.001). Mean CDT scores were also higher in S2 than S4 (*p* < 0.001) and S5 (*p* = 0.018). Mean CDT scores were higher in S3 than S2 (*p* < 0.001), S4 (*p* < 0.001), and S5 (*p* < 0.001). The mean CDT score in S5 was also higher than in S4 (*p* < 0.001). S1 and S3 scores were not significantly different (*p* = 0.066) (Figure 3).

**Figure 4:**
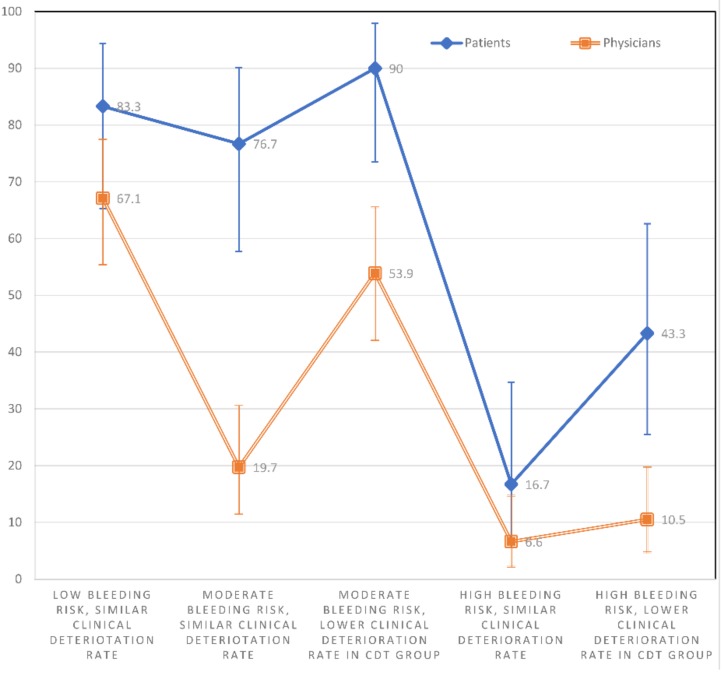
The percentage with 95% confidence intervals of physicians and patients showing a preference for CDT (score > 3) within each individual scenario.

A significantly greater percentage of patients than physicians showed a preference (score > 3) for CDT in scenarios S2 (76.7% vs. 19.7%; *p* < 0.001), S3 (90% vs. 53.9%; *p* = 0.002), and S5 (43.3% vs. 10.5%; *p* < 0.001); (Figure 4). In the other scenarios, patients tended to prefer CDT to a greater extent than physicians (NS), without reaching statistical significance. 

The percentages of patients who preferred CDT (score > 3) were higher in S1 than S4 (*p* < 0.001) and S5 (*p* = 0.002). The percentages were significantly higher in S2 than S4 (*p* < 0.001) and S5 (*p* = 0.013). The percentages were significantly higher in S3 than S4 (*p* < 0.001) and S5 (*p* < 0.001). They were also higher in S5 than S4 (*p* = 0.008). There was no significant difference in the percentages of patients who preferred CDT between S1 and S2 (*p* = 0.500), S1 and S3 (*p* = 0.625), or S2 and S3 (*p* = 0.219) (Figure 4).

The percentages of physicians who preferred CDT were higher in S1 compared to S2 (*p* < 0.001), S4 (*p* < 0.001), and S5 (*p* < 0.001). The percentages were higher in S3 than S4 (*p* < 0.001) and S5 (*p* < 0.001). Finally, a higher percentage of physicians preferred CDT in S3 than S2 (*p* < 0.001) and S2 than S4 (*p* = 0.013). There was no significant difference in the percentages of physicians who preferred CDT between S1 and S3 (*p* = 0.121), S2 and S5 (*p* = 0.118), or S4 and S5 (*p* = 0.453) (Figure 4).

In each scenario, there was no statistically significant difference in the CDT score between patients who had prior PE and those who did not (Table 2).

The results of the least squares regression analyses show that bleeding risk, clinical deterioration risk, and respondent type (patient or physician) were independent predictors of mean CDT scores (*p* < 0.001) when controlling for the other two factors. The effect of increased bleeding risk on CDT scores was unchanged at different levels of clinical deterioration risk and vice versa for both patients (*p* = 0.985) and physicians (*p* = 0.259). The effect of increased bleeding (*p* = 0.116) or clinical deterioration risk (*p* = 0.513) on CDT scores was not affected by responder type. Patient age, gender, and history of PE had no significant effect on CDT scores (*p* = 0.083, *p* = 0.071, *p* = 0.257 respectively). It was found that 60% of patients ≥ 60 years and 66% of patients < 60 years preferred CDT across all scenarios (NS, *p* = 0.601), and 66% of women and 57% of men preferred CDT across all scenarios (NS, *p* = 0.312).

Similarly, bleeding risk, clinical deterioration risk, and responder type were independent predictors of CDT preference (*p* < 0.001) when controlling for the other two factors. The effect of increased bleeding risk on CDT scores was unchanged at different levels of clinical deterioration risk and vice versa (*p* = 0.695). The effect of increased bleeding (*p* = 0.853) or clinical deterioration risk (*p* = 0.668) on CDT scores was also not affected by responder type. 

## 4. Discussion 

Since the passage of the Affordable Care Act and the creation of the Patient-Centered Outcomes Research Institute (PCORI), patient engagement in clinical trial design and decision making has gained traction [19]. Research funded by PCORI has shown that this shared approach results in better outcomes for patients with chest pain and prostate cancer [20,21]. At present, differing medical opinions and the lack of level 1 data for CDT have hampered shared decision making for this therapy in submassive PE [16]. An RCT of CDT reporting short and long-term outcomes would potentially improve decision making, but only if the results were relevant to all stakeholders. Thus, understanding how both patients and physicians would interpret the results of such a trial has considerable value. In this investigation, we sought to understand how physicians and patients would balance positive long-term trial results with varying risks of bleeding and clinical deterioration. We found that the majority of respondents would choose CDT if it improves long-term exercise capacity and does not increase bleeding. Patients appeared to accept a higher bleeding risk than physicians if CDT improved long-term exercise capacity.

The differential response to increased bleeding has several potential explanations. Physician respondents may be strongly influenced by their experience managing serious bleeding, and few physicians use thrombolytic therapy even in massive PE [22]. Moreover, most physicians who treat acute PE may not be personally involved in long-term follow-up and are therefore unfamiliar with long-term exercise limitation and reduced quality of life following PE [5,23]. On the other hand, patient respondents may not fully appreciate serious bleeding, or they accept the relatively low risk of serious bleeding if there is a high chance of maintaining good exercise capacity. A prospective observational study by Devereaux et al. showed that patients with atrial fibrillation placed more value on the avoidance of stroke and less value on the risk of bleeding than physicians [24]. Two similar studies incorporating patients’ preferences into decision making reached a similar conclusion [25,26]. In a systematic analysis of patient preferences of oral anticoagulants for stroke, Wilke et al. showed that patients were willing to accept 4.4 major bleeding risk to prevent one stroke [27]. In these studies, and ours, it appears that long-term disability is weighted heavily by patients, and major bleeding, while a modifier, is a risk they are willing to take in order to preserve quality of life.

Clinical deterioration also influenced patients and physicians differently. In scenarios with intermediate bleeding risk, more than 75% of patients preferred CDT, regardless of whether it decreased the rate of clinical deterioration. In contrast, the rate of clinical deterioration did affect physicians’ CDT preference in these scenarios: A majority of physicians preferred CDT only when the increased bleeding risk was offset by a decrease in clinical deterioration. These differences may also be the result of experience. Most physicians have witnessed and managed clinically deteriorating patients, whereas most patients do not have a personal understanding of such incidents.

The physician respondents were representative of real-world PE management teams. However, emergency medicine (EM) physicians were not represented. Though EM physicians are often involved in institutional PERTs, they may be under-represented in the PERT Consortium, or they declined to respond to the survey for unknown reasons [28]. The patient respondents were demographically similar to patients presenting with PE: More than 60% of patients were older than 60 years. It is likely that the NATF respondents are more informed about PE and its short and long-term consequences than other laypersons, given their active membership in a VTE patient-advocate organization. Therefore, their answers may differ from non-physicians who are not aware of PE.

There was no correlation between patients’ CDT scores or preference and age. These findings imply that patients value the ability to exercise regardless of age. Similarly, there was no correlation between CDT preference and history of prior PE. Conceivably, respondents who did not have a PE had a friend or family member who did, and this second-hand experience may explain the similar CDT scores between the 2 subgroups (PE vs. no-PE). There was also no association between patients’ gender and CDT scores, which is expected given that there are few clinically significant differences in the presentation, treatment, or clinical course of PE between genders [29,30]. However, these results need to be interpreted with caution, since the sample size was insufficient to allow us to perform well-powered subgroup analyses.

The present study has several limitations. First, although the survey was sent to each group twice, the response rate was low. Seventy-six physicians responded, providing a sufficient sample size (62 were required). However, only 30 NATF members responded, providing an insufficient sample size (64 were required). A better response rate may have been achieved by sending a third reminder and keeping the survey open for a longer period of time (not done for practical reasons). Administering the survey during annual meetings of the NATF and PERT consortium would theoretically improve the response rate, but these meetings did not occur during the time window of this survey. Second, there were likely selection biases. Surveys were sent to PERT Consortium physicians who are familiar with PE management and the current literature. Thus, their answers may not be generalizable. The same applies to the answers from the NATF community. These members have expressed a dedication to fighting PE, which may skew their preferences towards actively reducing PE-related morbidity. However, understanding the preferences of a group of patients and family members personally familiar with the morbidity associated with PE may be more useful than surveying a less-well-informed, general population. A third limitation is that the absolute positive control (improved exercise capacity and lower clinical deterioration) was not surveyed, though the relative contribution of such a scenario to the final results is likely minimal. Finally, differences in wording between the physician and patient surveys and varying levels of comprehension among patients may have influenced results. However, the concordance between patient and physician answers among scenarios (though the absolute percentages within scenarios differed) suggests internal validity.

## 5. Conclusions

The survey data indicate that both patients and physicians would choose CDT if it improved long-term exercise capacity and did not increase the risk of bleeding. The risk of bleeding and the extent of clinical deterioration are independent drivers of physician decision-making. Patients appear to accept a higher risk of bleeding to maintain exercise capacity. Based on these findings, a submassive PE RCT comparing CDT to anticoagulation alone with exercise capacity as the long-term primary endpoint would impact patient and physician decision-making, as long as short-term safety and efficacy were also examined. 

## Figures and Tables

**Table 1:** Scenarios presented to physicians and patients.

Scenario	Scenario Presented to Physicians	Scenario Presented to Patients	Summary
S1	The 6-minute walk distance is 40 m longer in the CDT group 7-day major and intracranial bleeding are equivalent in the 2 groups (2.5% major and 0.5% intracranial) 30-day clinical deterioration is equivalent in the 2 groups	Patients who get CDT + blood thinners are able to walk farther 1 year later than patients who only received blood thinners The risk of serious bleeding is the same in both groups (2.5%) The rates of intubation and chest compressions are the same in both groups	No difference in bleeding or clinical deterioration
S2	The 6-minute walk distance is 40 m longer in the CDT group 7-day major and intracranial bleeding are doubled in the CDT group, but still lower than the systemic thrombolysis group in PEITHO (5% major and 1% intracranial) 30-day clinical deterioration is equivalent in the 2 groups	Patients who get CDT + blood thinners are able to walk farther 1 year later than patients who only received blood thinners The rate of serious bleeding is moderately higher in patients who get CDT (risk is doubled). The rates of intubation and chest compressions are the same in both groups	The risk of major bleeding is doubled with CDT; no difference in clinical deterioration
S3	The 6-minute walk distance is 40 m longer in the CDT group 7-day major and intracranial bleeding are doubled in the CDT group, but still lower than the systemic thrombolysis group in PEITHO (5% major and 1% intracranial) 30-day clinical deterioration is lower in the CDT group	Patients who get CDT + blood thinners are able to walk farther 1 year later than patients who only received blood thinners The rate of serious bleeding is moderately higher in patients who get CDT (risk is doubled). Patients who get CDT + blood thinners do not have to be intubated or receive chest compressions as often as those who get blood thinners alone	The risk of major bleeding is doubled with CDT; clinical deterioration is lower in the CDT group
S4	The 6-minute walk distance is 40 m longer in the CDT group 7-day major and intracranial bleeding are 4 times higher in the CDT group, similar to the systemic thrombolysis group in PEITHO (10% major and 2% intracranial) 30-day clinical deterioration is equivalent in the 2 groups	Patients who get CDT + blood thinners are able to walk farther 1 year later than patients who only get blood thinners The rate of serious bleeding is much higher in patients who get CDT (risk is quadrupled). The rates of intubation and chest compressions are the same in both groups	The risk of major bleeding is quadrupled with CDT; no difference in clinical deterioration.
S5	The 6-minute walk distance is 40 m longer in the CDT group 7-day major and intracranial bleeding are 4 times higher in the CDT group, similar to the systemic thrombolysis group in PEITHO (10% major and 2% intracranial) 30-day clinical deterioration is lower in the CDT group	Patients who get CDT + blood thinners are able to walk farther 1 year later than patients who only received blood thinners The rate of serious bleeding is much higher in patients who get CDT (risk is quadrupled). Patients who get CDT + blood thinners do not have to be intubated or receive chest compressions as often as those who get blood thinners alone	The risk of major bleeding is quadrupled with CDT; clinical deterioration is lower in the CDT group

CDT, catheter-directed thrombolysis.

**Table 2:** The mean, standard deviation (SD), median, and inter-quartile range (IQR) of the ordinal scores for each scenario from patients with and without prior PE.

Scenario	No Prior PE (*n* = 14)				Prior PE (*n* = 16)				*p* Value
	Mean	SD	Median	IQR	Mean	SD	Median	IQR	
1	4.50	0.76	5.00	1.00	4.25	0.77	4.00	1.00	0.389
2	3.93	0.83	4.00	0.50	3.75	0.93	4.00	0.75	0.664
3	4.36	0.84	5.00	1.25	4.38	0.50	4.00	1.00	0.764
4	2.79	0.80	3.00	1.00	2.50	0.97	2.00	1.00	0.333
5	3.29	0.99	3.00	1.00	3.13	0.96	3.00	1.75	0.741

PE, pulmonary embolism.

## References

[1] Sista A.K., Horowitz J.M., Goldhaber S.Z. (2016). Four key questions surrounding thrombolytic therapy for submassive pulmonary embolism. Vasc. Med..

[2] Sista A.K., Goldhaber S.Z., Vedantham S., Kline J.A., Kuo W.T., Kahn S.R., Kabrhel C., McLaughlin V.V., White S.B., Kim N.H. (2016). Research priorities in submassive pulmonary embolism: Proceedings from a multidisciplinary research consensus panel. J. Vasc. Interv. Radiol..

[3] Klok F.A., Tijmensen J.E., Haeck M.L., van Kralingen K.W., Huisman M.V. (2008). Persistent dyspnea complaints at long-term follow-up after an episode of acute pulmonary embolism: Results of a questionnaire. Eur. J. Intern. Med..

[4] Meyer G., Vicaut E., Danays T., Agnelli G., Becattini C., Beyer-Westendorf J., Bluhmki E., Bouvaist H., Brenner B., Couturaud F. (2014). Fibrinolysis for patients with intermediate-risk pulmonary embolism. N. Engl. J. Med..

[5] Sista A.K., Klok F.A. (2017). Late outcomes of pulmonary embolism. The post-PE syndrome. Thromb. Res..

[6] Klok F.A., van der Hulle T., den Exter P.L., Lankeit M., Huisman M.V., Konstantinides S. (2014). The post-PE syndrome: A new concept for chronic complications of pulmonary embolism. Blood Rev..

[7] Chatterjee S., Chakraborty A., Weinberg I., Kadakia M., Wilensky R.L., Sardar P., Kumbhani D.J., Mukherjee D., Jaff M.R., Giri J. (2014). Thrombolysis for pulmonary embolism and risk of all-cause mortality, major bleeding, and intracranial hemorrhage: A meta-analysis. JAMA.

[8] Kuo W.T. (2012). Endovascular therapy for acute pulmonary embolism. J. Vasc. Interv. Radiol..

[9] Banovac F., Buckley D.C., Kuo W.T., Lough D.M., Martin L.G., Millward S.F., Clark T.W., Kundu S., Rajan D.K., Sacks D., Cardella J.F. (2010). Reporting standards for endovascular treatment of pulmonary embolism. J. Vasc. Interv. Radiol..

[10] Kearon C., Akl E.A., Comerota A.J., Prandoni P., Bounameaux H., Goldhaber S.Z., Nelson M.E., Wells P.S., Gould M.K., Dentali F. (2012). Antithrombotic therapy for VTE disease: Antithrombotic therapy and prevention of thrombosis, 9th ed: American College of Chest Physicians evidence-based clinical practice guidelines. Chest.

[11] Kearon C., Akl E.A., Ornelas J., Blaivas A., Jimenez D., Bounameaux H., Huisman M., King C.S., Morris T.A., Sood N. (2016). Antithrombotic therapy for VTE disease: CHEST guideline and expert panel report. Chest.

[12] Kucher N., Boekstegers P., Muller O.J., Kupatt C., Beyer-Westendorf J., Heitzer T., Tebbe U., Horstkotte J., Muller R., Blessing E. (2014). Randomized, controlled trial of ultrasound-assisted catheter-directed thrombolysis for acute intermediate-risk pulmonary embolism. Circulation.

[13] Kuo W.T., Banerjee A., Kim P.S., DeMarco F.J., Levy J.R., Facchini F.R., Unver K., Bertini M.J., Sista A.K., Hall M.J. (2015). Pulmonary embolism response to fragmentation, embolectomy, and catheter thrombolysis (PERFECT): Initial results from a prospective multicenter registry. Chest.

[14] Piazza G., Hohlfelder B., Jaff M.R., Ouriel K., Engelhardt T.C., Sterling K.M., Jones N.J., Gurley J.C., Bhatheja R., Kennedy R.J. (2015). A prospective, single-arm, multicenter trial of ultrasound-facilitated, catheter-directed, low-dose fibrinolysis for acute massive and submassive pulmonary embolism: The SEATTLE II study. JACC Cardiovasc. Interv..

[15] Hennemeyer C., Khan A., McGregor H., Moffett C., Woodhead G. (2019). Outcomes of catheter-directed therapy plus anticoagulation versus anticoagulation alone for submassive and massive pulmonary embolism. Am. J. Med..

[16] Taslakian B., Chawala D., Sista A.K. (2017). A survey of submassive pulmonary embolism treatment preferences among medical and endovascular physicians. J. Vasc. Interv. Radiol..

[17] Sullivan G.M., Artino A.R. (2013). Analyzing and interpreting data from likert-type scales. J. Grad Med. Educ..

[18] Jamieson S. (2004). Likert scales: How to (ab)use them. Med. Educ..

[19] Frank L., Basch E., Selby J.V. (2014). The PCORI perspective on patient-centered outcomes research. Jama.

[20] Hess E.P., Hollander J.E., Schaffer J.T., Kline J.A., Torres C.A., Diercks D.B., Jones R., Owen K.P., Meisel Z.F., Demers M. (2016). Shared decision making in patients with low risk chest pain: Prospective randomized pragmatic trial. BMJ.

[21] Chen R.C., Basak R., Meyer A.M., Kuo T.M., Carpenter W.R., Agans R.P., Broughman J.R., Reeve B.B., Nielsen M.E., Usinger D.S. (2017). Association between choice of radical prostatectomy, external beam radiotherapy, brachytherapy, or active surveillance and patient-reported quality of life among men with localized prostate cancer. Jama.

[22] Stein P.D., Matta F. (2012). Thrombolytic therapy in unstable patients with acute pulmonary embolism: Saves lives but underused. Am. J. Med..

[23] Kahn S.R., Hirsch A.M., Akaberi A., Hernandez P., Anderson D.R., Wells P.S., Rodger M.A., Solymoss S., Kovacs M.J., Rudski L. (2017). Functional and exercise limitations after a first episode of pulmonary embolism: Results of the ELOPE Prospective Cohort Study. Chest.

[24] Devereaux P.J., Anderson D.R., Gardner M.J., Putnam W., Flowerdew G.J., Brownell B.F., Nagpal S., Cox J.L. (2001). Differences between perspectives of physicians and patients on anticoagulation in patients with atrial fibrillation. observational study. BMJ.

[25] Protheroe J., Fahey T., Montgomery A.A., Peters T.J. (2000). The impact of patients’ preferences on the treatment of atrial fibrillation. observational study of patient based decision analysis. BMJ.

[26] Thomson R., Parkin D., Eccles M., Sudlow M., Robinson A. (2000). Decision analysis and guidelines for anticoagulant therapy to prevent stroke in patients with atrial fibrillation. Lancet.

[27] Wilke T., Bauer S., Mueller S., Kohlmann T., Bauersachs R. (2017). Patient preferences for oral anticoagulation therapy in atrial fibrillation. A systematic literature review. Patient.

[28] Barnes G.D., Kabrhel C., Courtney D.M., Naydenov S., Wood T., Rosovsky R., Rosenfield K., Giri J. (2016). Diversity in the pulmonary embolism response team model. An organizational survey of the National PERT Consortium members. Chest.

[29] Robert-Ebadi H., Le Gal G., Carrier M., Couturaud F., Perrier A., Bounameaux H., Righini M. (2010). Differences in clinical presentation of pulmonary embolism in women and men. J. Thromb. Haemost..

[30] Marshall A.L., Bartley A.C., Ashrani A.A., Pruthi R.K., Durani U., Gonsalves W.I., Kapoor P., Hashmi S.K., Siddiqui M.A., Go R.S. (2017). Sex-based disparities in venous thromboembolism outcomes: A National Inpatient Sample (NIS)-based analysis. Vasc. Med..

